# IL-17A Modulates Oxidant Stress-Induced Airway Hyperresponsiveness but Not Emphysema

**DOI:** 10.1371/journal.pone.0058452

**Published:** 2013-03-11

**Authors:** Mariona Pinart, Min Zhang, Feng Li, Farhana Hussain, Jie Zhu, Coen Wiegman, Bernard Ryffel, Kian Fan Chung

**Affiliations:** 1 Airways Disease, National Heart & Lung Institute, Imperial College, London, United Kingdom; 2 Université d’Orléans and CNRS, UMR 6218 Molecular Immunology and Embryology, Orléans, France; Chang Gung University, Taiwan

## Abstract

IL-17A induces the release of pro-inflammatory cytokines and of reactive oxygen species which could lead to neutrophilic inflammation. We determined the role of IL-17 receptor (IL-17R) signalling in oxidant-induced lung emphysema and airway hyperresponsiveness. IL-17R^−/−^ and wild-type C57/BL6 mice were exposed to ozone (3 ppm; 3 hours) for 12 times over 6 weeks. Bronchial responsiveness to acetylcholine was measured, and lungs were retrieved. Mean linear intercept (L_m_) and isometric contractile responses of intrapulmonary airways to acetylcholine were determined. In wild-type mice but not in IL-17R^−/−^, chronic ozone exposure caused airway hyperresponsiveness. The increase in L_m_ after chronic ozone exposure of wild-type mice was also observed in IL-17R^−/−^ mice. The increased maximal contractile response to acetylcholine seen in airways of wild-type mice exposed to ozone was abolished in IL-17R^−/−^ mice. p38-mitogen-activated protein kinase (MAPK) and dexamethasone-dependent increase in contractile response was reduced in airways from IL-17R^−/−^ ozone-exposed mice. Lung inflammation scores were not altered in IL-17R^−/−^ mice exposed to ozone compared to wild-type mice. The increased release of IL-17 and IL-1β, and the activation of p38 MAPK in the lungs of ozone-exposed mice was reduced in IL-17R^−/−^ mice. IL-17R signalling underlies the increase in airway hyperresponsiveness seen after ozone exposure, mediated by the increased contractility of airway smooth muscle. The emphysema and lung inflammation induced by ozone is not dependent on IL-17.

## Introduction

Cigarette smoking is the most commonly encountered risk factor for chronic obstructive pulmonary disease (COPD) and is a potent inducer of oxidative stress, which plays an important role in the pathogenesis of COPD by activating pathways that lead to chronic inflammation and emphysema as demonstrated in mouse models of cigarette exposure [1], [2]. Further evidence for a role for oxidative stress comes from the observation that, following cigarette smoke exposure, Nrf-2 knockout mice that express lower levels of antioxidant gene expression, were more susceptible to developing emphysema and lung inflammation [3].The crucial role of oxidant stress in the induction of COPD and emphysema is also supported by the observation that chronic exposure of mice to ozone, a ubiquitous oxidizing and toxic air pollutant generated photochemically from nitric oxides and hydrocarbons, led to the development of emphysema-like lung injury with alveolar enlargement and chronic lung inflammation [4]. In addition, exposure to ozone also increases the contractility of the airways and causes bronchial hyperrresponsiveness to constrictor agents such as acetylcholine [5].

Interleukin-17 (IL-17, also known as IL-17A), is produced by CD4+ Th17 cell, cytotoxic T-cells, invariant natural killer T-cells, lymphoid tissue-induced cells and γδT cells [6]. The IL-17 receptor (IL-17R) family comprises five receptor subunits, of which IL-17RA is the largest member and is necessary for IL-17A-mediated signal transduction [7]. IL-17A induces the release of the pro-inflammatory cytokines, CXCL-8, CXCL1 (GRO-α), KC, G-CSF and GM-CSF from airway epithelial cells, smooth muscle cells and macrophages, and thereby orchestrates neutrophilic inflammation and release of reactive oxygen species [6], [8].

The role of IL-17 in COPD has been reinforced by the report that over-expression of IL-17 in murine lung epithelium induced a COPD-like phenotype [9]. Administration of IL-17A into the airways increased neutrophil and chemokine expression [10]. IL-17A^+^ cells in the submucosa and IL-17 levels in the sputum were reported to be increased in COPD patients [11]. These observations indicate that IL-17 may play a role in COPD. Indeed, in a recent study of cigarette exposure in mice, the induction of emphysema was found to be partly dependent on IL-17 [12]. The induction of airway hyperresponsiveness by ozone exposure has also been shown to be dependent on IL-17 [13]. However, the role of IL-17 on the inflammatory response and emphysema induced by ozone are not known. We hypothesised that IL-17A may play an important role in airway hyperresponsiveness, pulmonary inflammation and emphysema induced by chronic exposure to ozone. We also studied the potential role of IL-17 on the direct contractile response of intrapulmonary airways to acetylcholine.

## Results

### In vivo Airway Responsiveness

**Figure 1:**
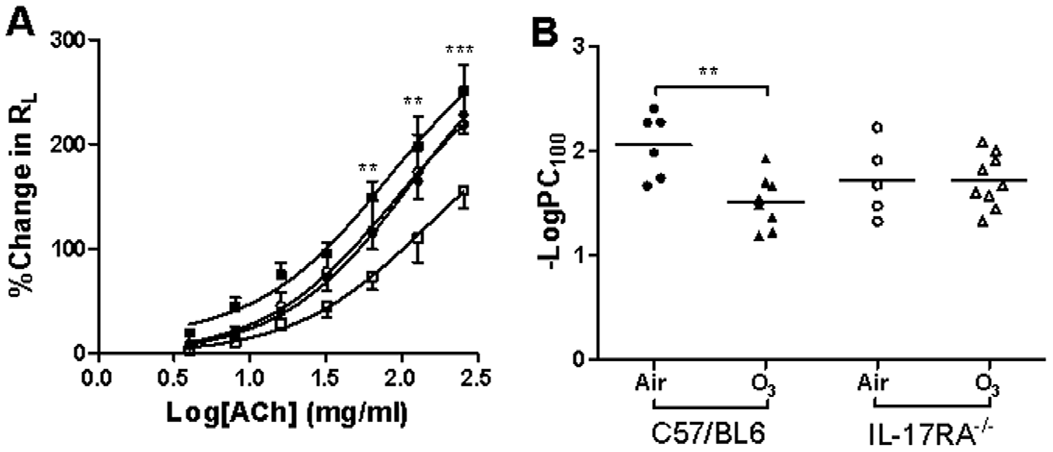
Concentration-response curves to acetylcholine (ACh) and –log provocative concentration of ACh required to increase lung resistance (R_L_) by 100% from baseline (PC_100_). C57/BL6 and IL-17R^−/−^ mice were exposed to air or to ozone. Data is expressed as mean ± S.E.M. **p<0.01; ***p<0.001, compared with to air-exposed mice.

There were no significant differences in the baseline lung resistance (R_L_) values following PBS challenge in the four groups of mice. Air-exposed IL-17R^−/−^ mice showed a non-significant higher responsiveness to ACh compared with air-exposed C57/BL6 mice (Fig. 1). Airway hyperresponsiveness (AHR) to ACh was induced in chronic ozone-exposed C57/BL6 mice compared with air-exposed mice (−logPC_100_ ozone: −1.510±0.088 vs. air: −2.055±0.126; p<0.01; Fig. 1). However, IL-17R^−/−^ mice exposed to ozone did not exhibit AHR to ACh compared with IL-17R^−/−^ air-exposed mice (–logPC_100_ ozone: −1.713±0.086 vs air: −1.722±0.160; Fig. 1).

### Isometric Contractile Response of Intrapulmonary Airways

**Figure 2:**
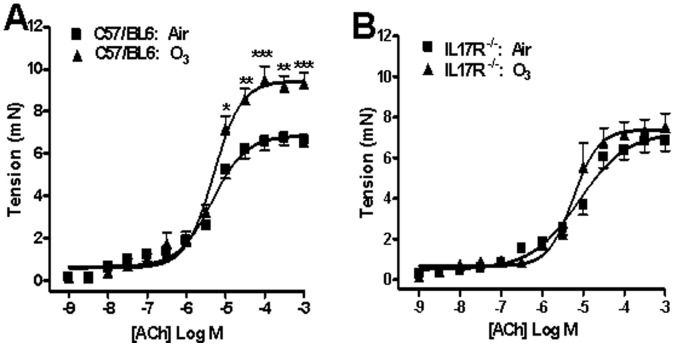
Acetylcholine (ACh)-induced isometric bronchial contractile tension. Air- and ozone-exposed C57/BL6 mice (6 in air- and 9 in ozone-exposed) and IL17RA^−/−^ mice (6 in air- and 5 in ozone-exposed) were studied. Data expressed as mean ±S.E.M. *p<0.05, **p<0.01, ***p<0.001, compared with air-exposed mice.

Isometric contractile responses to ACh (i.e E_max_) were similar in C57/BL6 mice and IL-17R^−/−^ mice exposed to air. E_max_ was increased in ozone-exposed C57/BL6 mice compared with air-exposed mice (9.44±0.28 vs 6.86±0.23 mN, respectively; p<0.05), but pEC_50_ values remained unchanged (5.30±0.06 vs 5.34±0.08) (Fig. 2A). However, E_max_ was not significantly changed in ozone-exposed IL-17R^−/−^ mice compared with air-exposed mice (7.39±0.30 vs 7.25±0.37 mN, respectively) and pEC_50_ values were not different (pEC_50_∶5.24±0.08 vs 5.33±0.11; Fig. 2B).

### Effects of p38-MAPK Inhibitor and Dexamethasone ex-vivo on Contractile Responses

**Figure 3:**
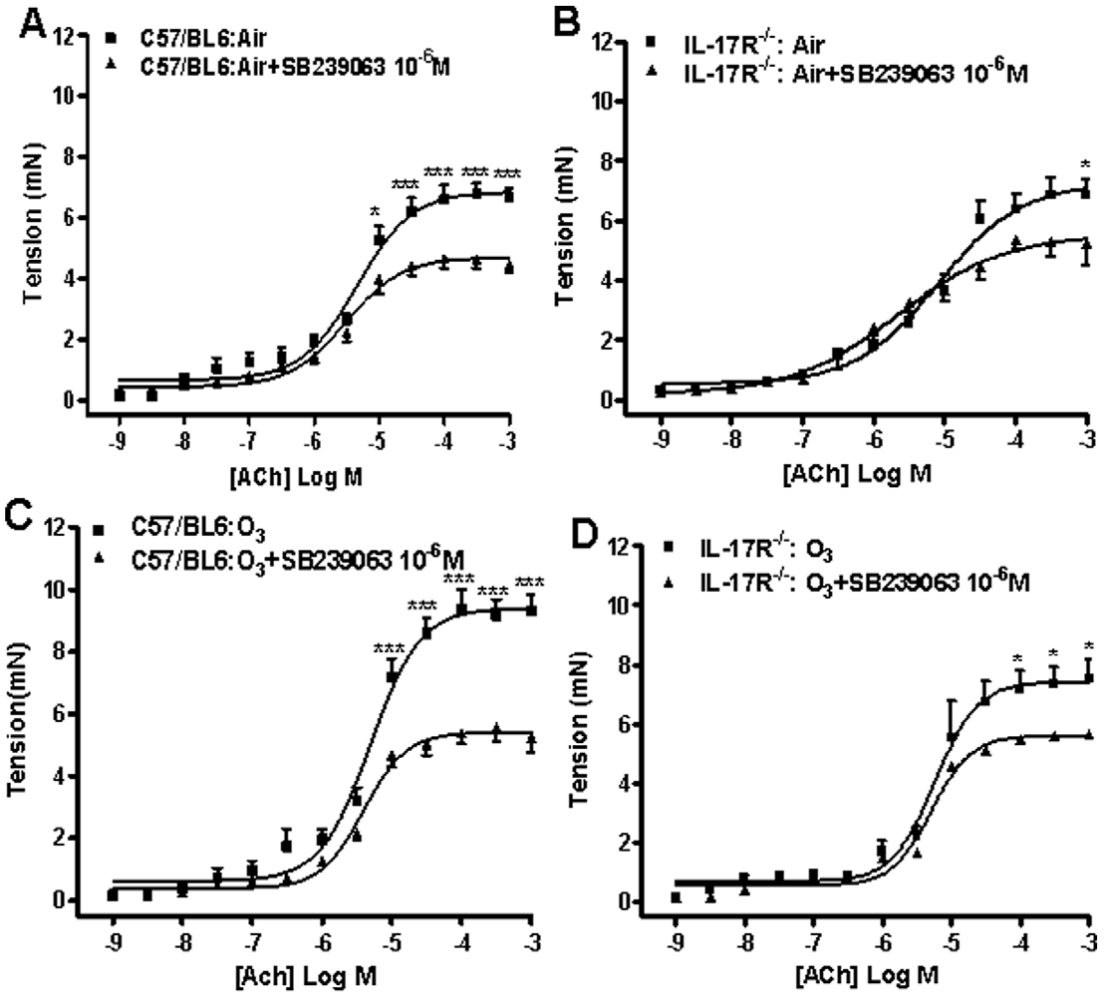
Effect of SB239063 (10^−6^ M) on acetylcholine (ACh)-induced bronchial contractile responses. Air-exposed C57/BL6 mice (n = 6; Panel A) and IL-17R^−/−^ mice (n = 6; Panel B) and ozone-exposed C57/BL6 mice (n = 9; Panel C) and IL-17R^−/−^ mice (n = 6; Panel D) were studied. Under each condition, the effect of SB239063 has been compared to responses in the absence of this inhibitor. Data presented as mean±SEM. *p<0.05; **p<0.01 compared with SB239063-treated tissues.

In order to understand how IL-17 can modulate the contractile process, we studied the contractile responses of the intrapulmonary airways in the presence of a selective p38 MAPK inhibitor, SB239063 (10^−6^ M), and of a corticosteroid, dexamethasone (10^−6^ M), as previously described [14]. In the presence of SB239063, there was a reduction in the maximal contractility response in both mouse strains exposed to air or to ozone. Comparing the airways from ozone–exposed mice from the C57/BL6 and IL-17R^−/−^ mice, we found a p38-dependent portion of the contractility attributable to IL-17 (Fig. 3C, D).

**Figure 4:**
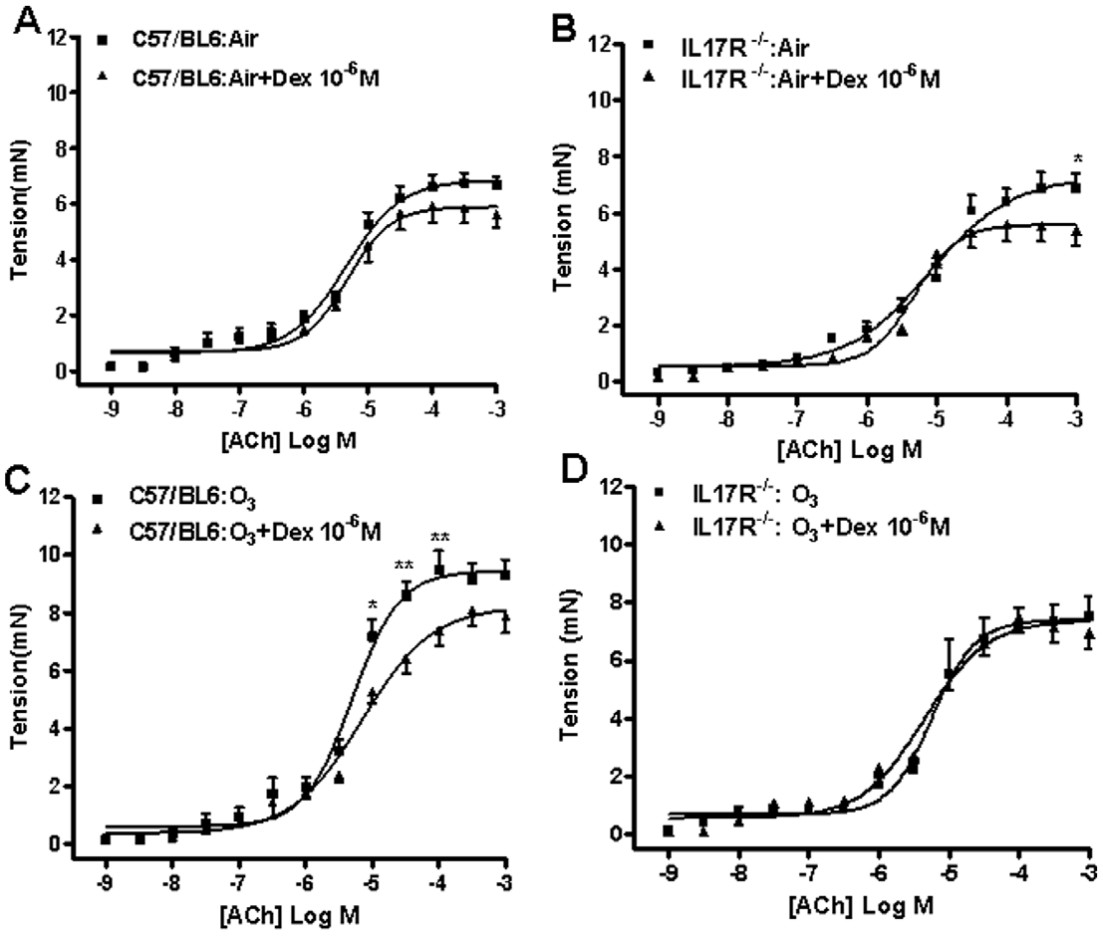
Effect of dexamethasone (10^−6^ M) on acetylcholine (ACh)-induced bronchial contractile responses. Air-exposed C57/BL6 mice (n = 6; Panel A) and IL-17R^−/−^ mice (n = 6; Panel B) and ozone-exposed C57/BL6 mice (n = 9; Panel C) and IL-17R^−/−^ mice (n = 6; Panel D) were studied. Under each condition, the effect of dexamethasone has been compared to responses in the absence of this inhibitor. Data presented as mean±SEM. *p<0.05 compared with dexamethasone-treated tissues.

We also studied the effect of dexamethasone on the contractile responses *ex-vivo*. In C57/Bl6 mice, dexamethasone inhibited the maximal contractile responses to acetylcholine non-significantly, although in IL-17R^−/−^ mice, this inhibition was significant in air-exposed mice (Fig. 4). The effect of dexamethasone on ozone-exposed airways was different in the C57/Bl6 and IL-17R^−/−^ mice. Hence, dexamethasone inhibited ozone-induced enhancement of the contractile response in C57/Bl6 mice but there was no such inhibition in IL-17R^−/−^ mice exposed to ozone.

### Mean Linear Intercept (L_m_ ) and Emphysema Scores

**Figure 5:**
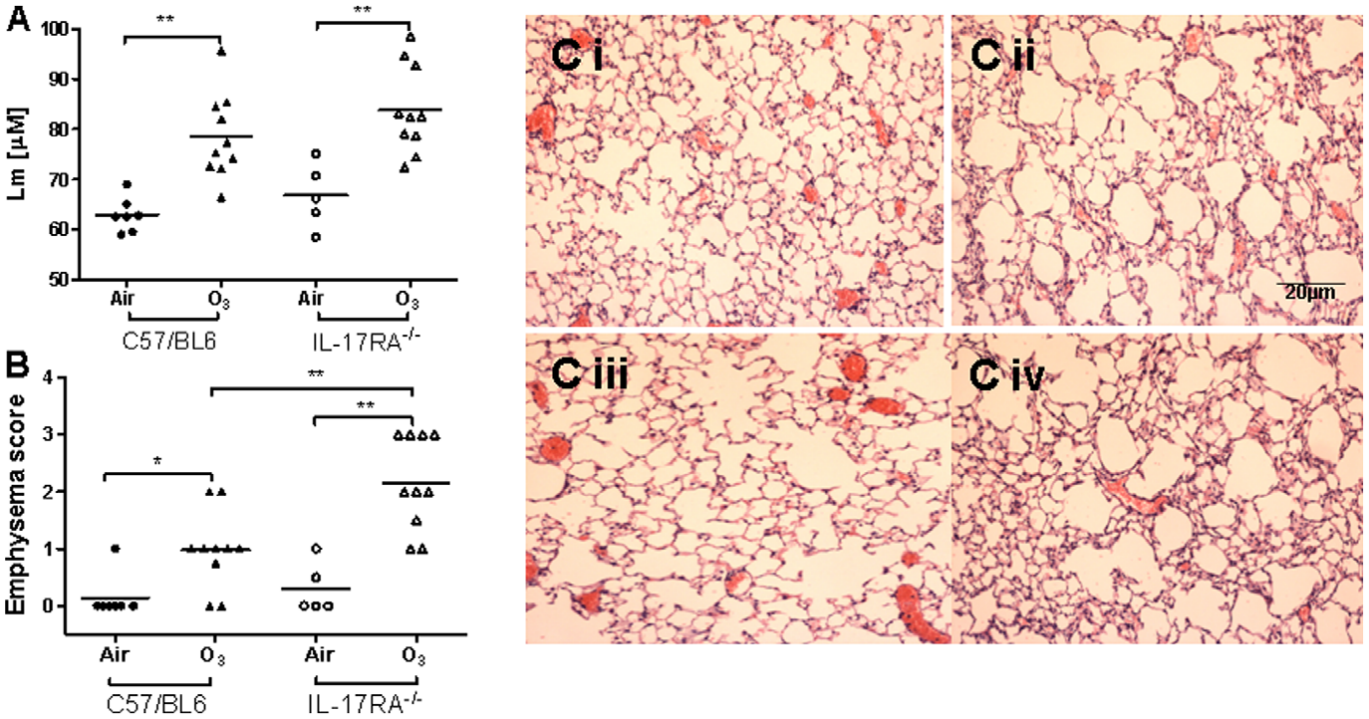
Mean linear intercept (L_m_) in the lungs of air- and ozone exposed mice (Panel A). Lungs inflated at 25 cm of water were sectioned and stained with haematoxylin and eosin and microscopically assessed for L_m_. Ozone exposed C57/BL6 mice and IL-17R^−/−^ mice showed increased Lm (alveolar enlargement) compared with their appropriate air-exposed control mice. Emphysema score in the lungs of air- and ozone exposed mice (Panel B). Compared with air exposed mice, the emphysema score was increased in ozone-exposed C57/BL6 and IL-17R^−/−^mice, while it was increased further in ozoneexposed IL-17R^−/−^ mice. Data are expressed as means ± SEM. *p<0.05; **p<0.01; ***p<0.001. Representative histological sections of mouse lungs (Panel C i, ii, iii & iv). Lung sections were stained with haematoxylin and eosin after 6 weeks of exposure to ozone showing enlargement of alveolar spaces in C57/BL6 (Panel C ii) and IL-17A^−/−^ mice (Panel C iv).

There was no significant difference in L_m_ in air-exposed C57/BL6 mice and IL-17R^−/−^ mice (50.31±1.67 vs 53.61±2.12). L_m_ was increased in ozone-exposed C57/BL6 mice compared with air-exposed mice (Fig. 5A), indicating that ozone-exposed mice showed an increase in alveolar size and therefore developed an emphysematous-like pattern (ozone: 66.79±2.78 vs air: 50.31±1.67, P<0.001). Similarly, L_m_ was increased in ozone-exposed IL-17R^−/−^ mice compared to air-exposed mice (ozone: 65.16±2.04 vs air: 53.61±2.12) (Fig. 5A).

The baseline emphysema score was similar in air-exposed C57/BL6 mice and IL-17R^−/−^ mice. The emphysema score was increased in both ozone-exposed C57/BL6 and IL-17R^−/−^mice. However, IL-17R^−/−^ mice exposed to ozone had a higher emphysema score compared with C57/BL6 mice (IL-17R^−/−^:2.15±0.26 vs C57/BL6∶0.98±0.21, p<0.01) (Fig. 4B). Representative photomicrographs in Fig. 5C show alveolar enlargement occurring in the ozone-exposed mice.

### Inflammation Scores

**Figure 6:**
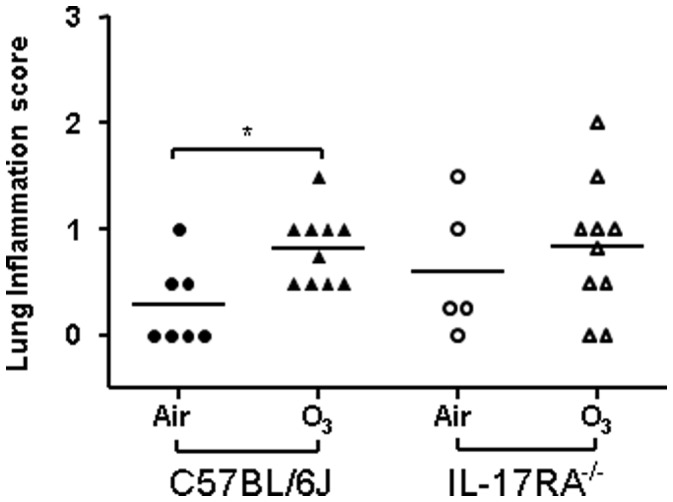
Inflammation score in the airways and lungs of air- and ozone-exposed mice. Compared with air exposed control mice, the inflammation score was increased significantly in ozone exposed C57/BL6 mice but not in IL-17R^−/−^mice. Data are expressed as means ± SEM. *p<0.01.

There was no significant difference in the lung inflammation score at baseline in the air-exposed C57/BL6 and IL-17R^−/−^ mice. In the IL-17R^−/−^ mice exposed to ozone, the inflammatory score was not significantly different from the air-exposed mice; however, this may be accounted for by the slightly higher baseline inflammation score in the IL-17R^−/−^ mice (Fig. 6). The inflammation score significantly increased in ozone-exposed C57/BL6 mice but not in IL-17R^−/−^ mice (Fig. 5).

### Lung IL-17A, IL-1β and TNFα and Phosphorylated p38, JNK and ERK

**Figure 7:**
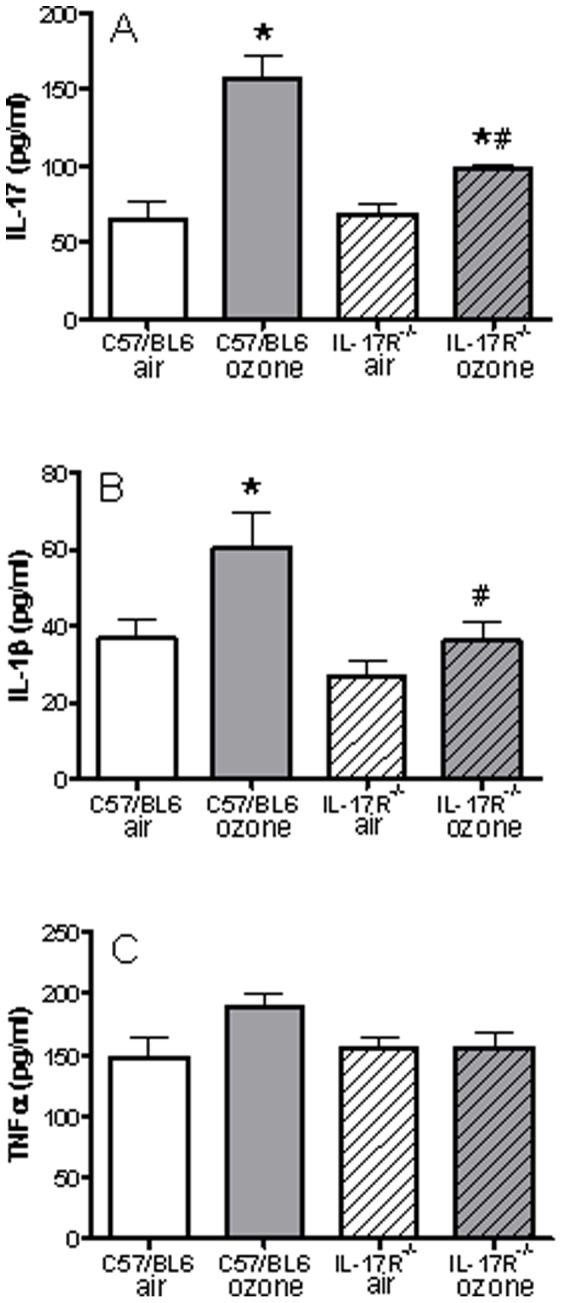
Levels of lung IL-17, IL-1β and TNFα. C57/Bl6 and IL-17R^−/−^ mice were exposed to air or to ozone. Data shown as mean ± SEM for n = 5 in each group; *p<0.05 compared to air in the same species; ^#^p<0.05 compared to C57/Bl6 mice with corresponding exposure.

In C57/Bl6 wild-type mice, lung levels of IL-17 were increased after ozone exposure (Fig. 7). Baseline levels of lung IL-17 in IL-17R^−/−^ mice were similar to those in C57/Bl mice but the levels of lung IL-17 were less increased after ozone exposure (Fig. 7). Similarly, lung levels of IL-1β increased in C57/Bl6 mice exposed to ozone, but these levels were reduced in IL-17R^−/−^ mice exposed to ozone. Lung levels of TNFα did not increase significantly after ozone exposure.

**Figure 8:**
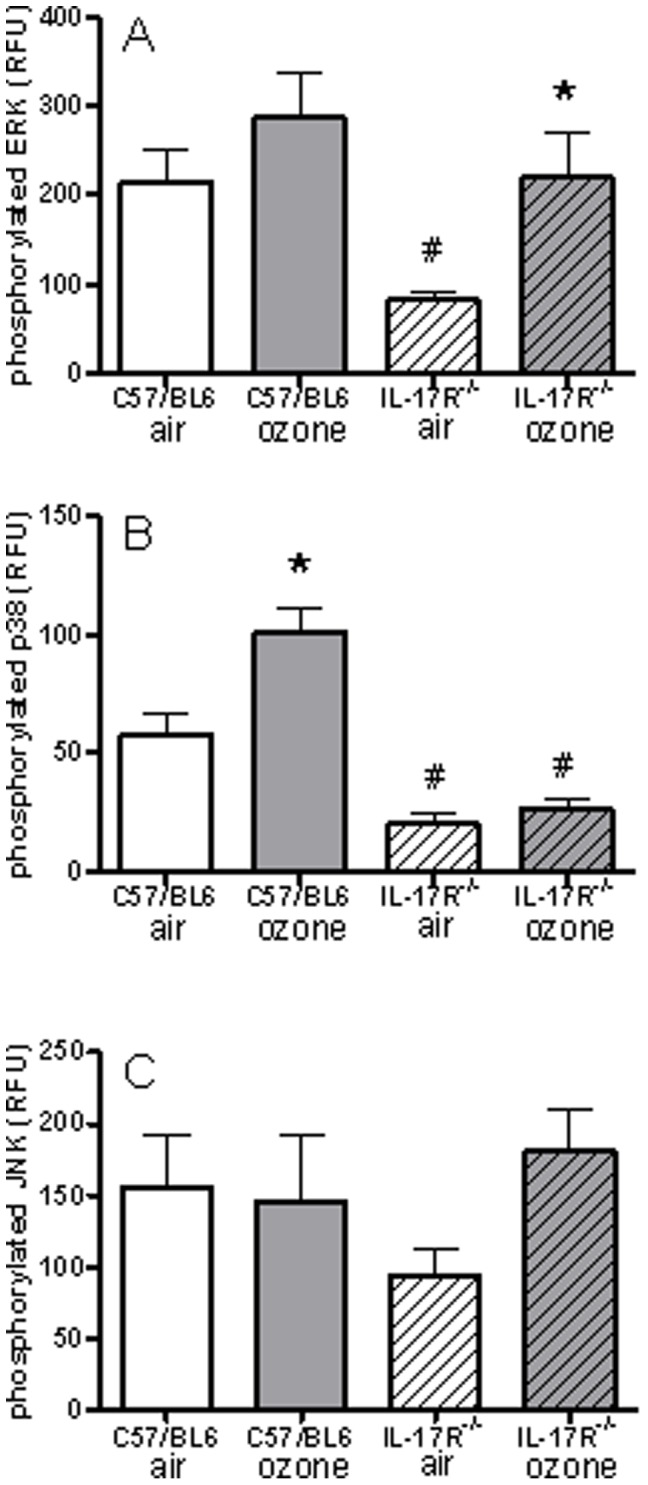
Lung levels of phosphorylated ERK1/2, p38 MAPK and JNK 1/2/3. C57/Bl6 and IL-17R^−/−^ mice were exposed to air or to ozone. Data shown as mean ± SEM for n = 5 in each group; *p<0.05 compared to air in the same species; ^#^p<0.05 compared to C57/Bl6 mice with corresponding exposure. RFU: Relative fluorescence unit.

In C57/Bl6 mice, ozone exposure also led to an activation of p38 MAPK but not of ERK or JNK, since the levels of phosphorylated p38 were increased. On the other hand, in IL-17R^−/−^ mice exposed to air, the amount of phosphorylated ERK and p38 measured was significantly reduced compared to C57/Bl6 mice. While ERK activity was increased by ozone exposure in IL-17R^−/−^ mice, p38 activation was completely suppressed (Fig. 8).

## Discussion

We have shown that repeated exposure to ozone induces lung emphysema and inflammation as previously reported, and in this study, we also demonstrated an increase in airway responsiveness to acetylcholine, accompanied by an *ex-vivo* increase in the maximal isometric contractile response to acetylcholine. The lung emphysema and inflammation, with the airway hyperresponsiveness are both irreversible when mice were studied up to 6 weeks after cessation of exposure to ozone (*unpublished data*). We now demonstrate that, in the IL-17 receptor knock-out mouse model, the airway hyperresponsiveness induced by multiple exposures to ozone is inhibited; we also found that this was accompanied by no enhancement of the maximal contractile response of bronchial airways. However, there was no inhibition of the emphysematous process and the chronic inflammatory response induced by chronic ozone exposure. Thus, we conclude that IL-17 is important for ozone-induced bronchial hyperresponsiveness but not for the induction of emphysema and inflammation. This dependent effect of airway hyperresponsiveness on IL-17 is likely to be a direct effect of IL-17 on airway smooth muscle. Our data is in agreement with previous studies using mice deficient in IL-17A or IL-17R. These studies reported that IL-17A was necessary for the development of airway hyperresponsiveness in an ovalbumin-induced asthma model [15], [16]. Furthermore, a recent study using IL-17 knock-out mice reported that IL-17 was required for ozone-induced airway hyperresponsiveness through stimulation of the production of IL-17 by i-NKT cells and T cells in the lungs [13].

The mechanisms of ozone-induced airway hyperresponsiveness are complex. Our previous work indicated that a single ozone exposure could directly cause an increase in airway smooth muscle contractility to acetylcholine, which may contribute to the pathogenesis of airway hyperresponsiveness. Direct instillation of IL-17 into the airways of ovalbumin-sensitized and -challenged mice could trigger robust airway hyperresponsiveness [17]. IL-17 has also been shown to activate many common downstream signalling pathways, including NF-κB, and the MAPKs (mitogen-activated protein kinases) JNK (c-Jun N-terminal kinase), p38 and ERK (extracellular-signal-regulated kinase), together with other kinases including PI3K (phosphoinositide 3-kinase) and JAK (Janus kinase)/STATs [7]. IL-17 may increase AHR e through a direct action on the airway smooth muscle as IL-17 has been shown to activate p38 MAPK pathway in these cells [18] . We have previously shown the p38 MAPK/HSP27 is involved in the *ex-vivo* cholinergic agonist-induced increase in bronchial smooth muscle contractility following a single exposure to ozone [14]. We now show a similar response in the airways from mice chronically-exposed to ozone, with the development of concomitant emphysema. Our ex-vivo data of airway smooth muscle contractility indicates that IL-17 may contribute partly to the activation of p38 MAPK underlying the increase in contractility induced by ozone. Furthermore, in the current study, we demonstrate that IL-17A and IL-1β are produced in the lung after chronic exposure to ozone, and interestingly, this production was dependent partly on the presence of IL-17 receptor. This is particularly interesting for IL-17, which indicates an autoregulatory mechanism on the production of IL-17 through its own receptor. In addition, p38 MAPK activation is demonstrated after ozone exposure also, and this was abolished in IL-17R^−/−^ mice. These observations in the lungs provide further support to the concept that IL-17 may contribute to the activation of p38 MAPK.

Another aspect of interest in our study is the fact that dexamethasone inhibition of the maximal contractile response in IL-17R^−/−^ mice is lost following exposure to chronic ozone exposure, an indication of corticosteroid insensitivity of the airway smooth muscle. This was not present in the wild-type mice, which indicates that IL-17A is important in determining the sensitivity of the airway smooth muscle to corticosteroids. This observation contrasts with the recent reports that IL-17 may be involved in the induction of corticosteroid insensitivity in human epithelial cells [19] or could be the causative link between Th17 cells and glucocorticosteroid-insensitive allergic airway hyperrresponsiveness in the mouse [20]. It is likely that these differences relate to our unique challenge model of chronic ozone model.

IL-17 has been implicated in promoting pulmonary inflammation and tissue destruction, specifically through upregulation of MMP-9 and its effects as a neutrophil chemoattractant [21]. IL-17 stimulates mucin production by respiratory epithelial cells [22]. As both neutrophilic infiltration and mucus hypersecretion are characteristics of COPD, Th17 cells may play an important role in promoting these responses. There is evidence that IL-17R^−/−^ mice failed to develop emphysema after 6 months of cigarette smoke exposure, indicating a role for IL-17 in the development of cigarette-smoke-induced emphysema [12]. Further evidence of the role for Th17 cells in cigarette smoke induced inflammation and airspace enlargement is the study showing that CCR6-positive Th17 cell population increased in bronchoalveolar lavage fluid of chronic smoke-exposed mice [11]. However, we were unable to observe any protective effect against ozone-induced emphysema in our IL-17R^−/−^ mice, indicating that a specific oxidative stress stimulus such as ozone does not require IL-17 or Th-17 cells to induce emphysema. We hypothesise that components of the complex mixture that is cigarette smoke apart from direct oxidant gases are capable of acting as a Th17 adjuvant to activate metalloproteases such as MMP-12. The inflammation score tended to be higher in air-exposed IL-17R^−/−^ mice compared with C57/BL6 mice, and was within the same range in both mouse species following chronic ozone exposure, leading us to conclude that the chronic lung inflammation is not IL-17 dependent.

In conclusion, in the chronic exposure model to an oxidant, ozone, the induced airway hyperresponsiveness and associated increase in maximal contractile responses to acetylcholine are dependent on IL-17, and this is likely to represent an IL-17-dependent activation of p38 MAPK. The induction of emphysema and lung inflammation in this ozone exposure model are not IL-17-dependent.

## Materials and Methods

### Mice and Ozone Exposure

Pathogen-free, 10–12 week old male C57/BL6 mice (Harlan, UK) and IL-17R-deficient (IL-17R^−/−^) mice were housed within ‘maximiser’ filter-topped cages (Maximiser, Theseus caging system Inc., Hazelton, PA, USA), maintained in a temperature-controlled (23°C) facility with a strict 12-h light–dark cycle and were given free access to food and water. IL-17R^−/−^ mice were on C57/BL6 genetic background, as previously described [23] and were obtained from Centre National de la Recherche Scientifique (CNRS), Orléans, France. The protocols were approved by the Imperial College Biosciences group and performed under a licence from the Home Office, UK, under the Animals (Scientific Procedures) Act 1986.

The two strains of mice (IL-17R^−/−^ and C57/BL6) were exposed to ozone produced from an ozoniser (Model 500 Sander Ozoniser, Germany), mixed with air, for 3 hours at a concentration 2.5 parts per million (ppm) in a sealed Perspex container. Mice received ozone twice a week for 6 weeks. Control animals received medical air only over the equivalent period. Ozone concentration was continuously monitored with an ozone probe (ATi Technologies, Ashton-U-Lyne, UK).

### Measurement of Airways Hyperresponsievness (AHR)

Twenty-four hours following the last ozone exposure, mice were anesthetized with an intraperitoneal injection of anesthetic solution containing midazolam (Roche Products Ltd., Welwyn Garden City, UK) and Hypnorm (0.315 mg/ml fentanyl citrate and 10 mg/ml fluanisone; Janssen Animal Health, Wantage, UK). Mice were tracheostomised and ventilated (MiniVent type 845, Hugo Sach Electronic, Germany; rate: 250 breaths/min and tidal volume: 250 µl), and were monitored in a whole body plethysmograph with a pneumotachograph connected to a transducer (EMMS, Hants, UK). Transpulmonary pressure was assessed via an esophageal catheter (EMMS, Hants, UK). Instantaneous calculation of pulmonary resistance (R_L_) was obtained. Increasing concentrations of acetylcholine (ACh) (Sigma, Dorset, UK) (4–256 mg/ml) were administered with an Aeroneb® Lab Micropump Nebulizer (EMMS, Hants, UK), and R_L_ was recorded for a 3-min period following each concentration. R_L_ after each concentration was expressed as percentage change from baseline R_L_ measured following nebulized PBS (Sigma, Dorset, UK). The concentration of acetylcholine required to increase R_L_ by 100% from baseline was calculated (PC_100_) and –log PC_100_ was taken as a measure of airway responsiveness.

### Bronchial Ring Preparation and Myography

The lung was dissected out whole following terminal anaesthesia with pentobarbitone. The inferior lobe of the right lung was rapidly excised and immersed in physiological salt solution (PSS; NaCl, 119 mM; KCl, 4.7 mM; CaCl_2_, 2.5 mM; MgSO_4_, 1.17 mM; NaHCO_3_, 25 mM; KH_2_PO_4_, 1.18 mM; EDTA, 0.027 mM; and glucose, 5.5 mM). Intrapulmonary bronchi were dissected under the microscope, parenchymal and connective tissues were carefully removed. Intact bronchi, 200–400 µm in diameter, 2 mm in length, were mounted on to metal prongs of a myograph (Myograph 610 M, Danish Myo Technology, Aarhus, Denmark), suspended in an organ bath, filled with 5 ml of PSS, bubbled with 95% oxygen and maintained at 37°C. Isometric tension was recorded and analysed using Chart software (AD Instruments Ltd., Hastings, U.K.). The optimal length for each bronchial ring was obtained through incremental radial stretch (passive tension) and repeated stimulation with 124 mM potassium PSS (active tension). Optimal length was taken at the point at which increased stretch ceased to increase active tension. Bronchi were then allowed to equilibrate for 30 min in PSS and 3 µM indomethacin was added into the organ bath to inhibit prostaglandin release. The bronchial contractile response in air- and ozone-exposed mice was generated with 10^−9^ M to 10^−3^ M of ACh. The concentration-response curves were fitted by nonlinear regression and with Hill equation (GraphPad Prism 4.03, San Diego, CA, USA) to provide an estimated maximal contraction (E_max_) and the negative logarithm of the effective concentration to cause 50% of the maximal contractile response (pEC_50_).

### Histological and Morphometric Analysis

The left lung was inflated with fresh 4% paraformaldehyde and maintained with 25 cm of water pressure for at least 4 hours and then embedded in paraffin. Paraffin blocks were sectioned to expose the maximum surface area of lung tissue in the plane of the bronchial tree. Five µm sections were cut and stained with haematoxylin and eosin (H&E). All counts were performed by observers who were blinded as to the mice studied.

The mean linear intercept, a measure of interalveolar septal wall distance, was determined using a reticule with a Thurlbeck grid comprising of 5 lines (each 550 µM long), with 10 fields per section assessed at random. Two slides per mouse were coded and analyzed. Fields with airways or vessels were avoided by moving one field in any one direction. L_m_ was calculated by dividing the length of the line by the number of alveolar wall and grid line interceptions.

The severity of emphysematous change observed in the H&E stained lung sections was also scored on a 0–3 scale defined as: 0 = no emphysema; <1 = lung parenchyma is involved; 1 = lung parenchyma is involved with small enlargement of alveolar space; 2 = lung parenchyma is involved with medium enlargement of alveolar space; 3 = lung parenchyma is involved with pronounced enlargement of alveolar space.

The severity of inflammatory response observed in the H&E stained lung sections was scored on a 0–3 scale defined as: 0 = no inflammatory response, 1 = mild inflammation with foci of inflammatory cells in bronchial or vascular wall and in alveolar septa; 2 = moderate inflammation with patchy inflammation or localised inflammation in walls of bronchi or blood vessel and alveolar septa and less than 1/3 of lung cross-sectional area is involved; and 3 = severe inflammation with diffuse inflammatory cells in walls of bronchi or blood vessels, and alveoli septa; between one third to two thirds of the lung area is involved.

#### Lung protein isolation, MAP kinase phosphorylation status and cytokines assays

Lung tissue (30 mg) was homogenised using 1.4 mm Precellys Ceramic beads and Precellys 24 homogeniser (Peqlab, Erlangen, Germany) at 6800 rpm for 15 seconds. Cytosolic proteins were extracted with a hypotonic buffer (Active Motif, part #100505) and detergent (Active motif, part #100512) by centrifugation at 14000 rpm for 30 seconds at 4°C. Final protein concentration was determined using a protein assay reagent kit (Pierce Chemical, Rockford, IL, USA) and BSA standard (Sigma, Gillingham, UK).

MAP kinase protein phosphorylation was determined in cytoplasmic protein extracts using the PhosphoTracer ERK1/2 (pT202/Y204)+p38 MAPK (pT180/Y182)+JNK1/2/3 (pT183/Y185) Elisa Kit (Abcam, Cambridge, UK). The kit detects ERK1 and 2, p38 and JNK1, 2 and 3 only when phosphorylated on the indicated conserved threonine or tyrosine sites of each protein and was used according to manufacturer’s instructions. Fluorescent data was normalised against total protein concentration from the same sample.

IL-17, IL-1β and TNFα concentrations were measured in lung homogenate supernatants with commercial available ELISA kits (R&D Systems Europe Ltd, Abingdon, UK) and were performed according to manufacturer’s instructions.

### Data Analysis

Data are presented as mean ± S.E.M. For multiple comparisons of different groups (% change in R_L_), two-way analysis of variance was performed. Unpaired t-test was carried out for comparison between two individual groups. For data that did not comply with the normality and homoscedasticity assumptions, nonparametric t-test (U-Mann Whitney) analyses were carried out. All hypothesis testing was two-sided and a p value of less than 0.05 was accepted as significant.
